# Combined deficiency of Notch1 and Notch3 causes pericyte dysfunction, models CADASIL, and results in arteriovenous malformations

**DOI:** 10.1038/srep16449

**Published:** 2015-11-13

**Authors:** Natalie M. Kofler, Henar Cuervo, Minji K. Uh, Aino Murtomäki, Jan Kitajewski

**Affiliations:** 1 Department of Ob/Gyn, Columbia University, New York, NY 10032, USA; 2 Division of Genetics, Biosciences, Viikki Biocenter, University of Helsinki, POB 56, FIN-00014, Helsinki, Finland; 3 Pathology, Columbia University, New York, NY 10032, USA; 4 Herbert Irving Comprehensive Cancer Center, Columbia University, 1130 St. Nicholas Ave, New York, NY 10032, USA

## Abstract

Pericytes regulate vessel stability and pericyte dysfunction contributes to retinopathies, stroke, and cancer. Here we define Notch as a key regulator of pericyte function during angiogenesis. In *Notch1*^+/−^; *Notch3*^−/−^ mice, combined deficiency of Notch1 and Notch3 altered pericyte interaction with the endothelium and reduced pericyte coverage of the retinal vasculature. Notch1 and Notch3 were shown to cooperate to promote proper vascular basement membrane formation and contribute to endothelial cell quiescence. Accordingly, loss of pericyte function due to Notch deficiency exacerbates endothelial cell activation caused by Notch1 haploinsufficiency. Mice mutant for Notch1 and Notch3 develop arteriovenous malformations and display hallmarks of the ischemic stroke disease CADASIL. Thus, Notch deficiency compromises pericyte function and contributes to vascular pathologies.

Pericytes and vascular smooth muscle cells (VSMCs), collectively referred to as mural cells, fulfill distinct roles in the vasculature. VSMCs lend support and contractility to large vessels, while capillary-associated pericytes promote endothelial quiescence and vessel maturation during sprouting angiogenesis^1^. Angiogenesis in the absence of pericytes results in vessel dilation, impaired capillary formation, and increased vessel permeability^2^,^3^,^4^,^5^. Pericyte dysfunction is a feature of vascular pathologies, where loss of retinal pericytes precedes histopathological features of diabetic retinopathy and pericyte dissociation is observed in the microvascular stroke disease CADASIL (cerebral autosomal dominant arteriopathy with subcortical infarcts and leukoencephalopathy)^6^,^7^.

During angiogenesis, pericyte recruitment parallels vessel outgrowth and depends on the platelet derived growth factor receptor (PDGFR)-β response to PDGF-B secreted by endothelial tip cells^8^,^9^. PDGFR-β activation in pericytes promotes their migration and proliferation. Inhibition of PDGFR-β prevents pericyte recruitment resulting in vascular defects^10^,^11^. Once recruited to nascent vessels, pericytes function to stabilize the microvasculature through induction of vascular basement membrane (vBM) formation. In the retina, lack of pericyte coverage alters the vBM and *in vitro* modeling of angiogenesis demonstrates that pericytes induce vBM deposition^2^,^12^.

Notch signaling has an established role in endothelial cell function and large vessel maturation^13^. Mammals express four Notch receptors (Notch1-4), which interact with five membrane-bound ligands, Jagged1, Jagged2, and delta-like ligand (Dll)-1, −3, and −4^14^. Upon ligand binding, the Notch intracellular domain is released from the plasma membrane by two proteolytic cleavage events, the final mediated by gamma-secretase. Following cleavage, the Notch intracellular domain translocates to the nucleus to form a complex with CSL (CBF-1, suppressor of Hairless, LAG-1)/RBPjκ and Mastermind-like proteins (MAML)^15^,^16^. This complex activates transcription of canonical Notch target genes, including Hes and Hey transcriptional repressors, PDGFR-β, and smooth muscle α-actin (αSMA)^17^,^18^,^19^,^20^. In endothelial cells, Notch1 responds to Dll4 causing restriction of the tip cell phenotype. Accordingly, mice heterozygous for Dll4 have hyper-vascularized retinas^21^,^22^.

Studies on Notch function in mammalian mural cells have largely focused on Notch3 in VSMCs lining arteries and arterioles. In humans, inherited mutations in NOTCH3 cause the lethal stroke disease CADASIL, characterized by mural cell dysfunction and loss of arterial vascular integrity^23^,^24^. Mice deficient for Notch3 display reduced arterial VSMC coverage and impaired VSMC maturation, yet they do not recapitulate a CADASIL phenoytpe^25^,^26^,^27^. In zebrafish, Notch3 has been implicated in assuring pericyte coverage and vessel integrity^28^. *In vitro* studies have shown that Notch signaling promotes pericyte survival and adhesion to endothelial cells^29^,^30^. A role for Notch1 in pericytes has yet to be described.

We hypothesized that both Notch1 and Notch3 are required for pericyte function in the vasculature. We assessed retinal angiogenesis in mice with combined deficiency of Notch1 and Notch3 and found that Notch signaling was required for proper pericyte coverage and interaction with the endothelium. Furthermore, we observed that both Notch1 and Notch3 are required for vascular basement membrane formation. Pericyte dysfunction due to Notch1 and Notch3 deficiency, and endothelial activation as a result of Notch1 haploinsufficiency caused retinal hyper-vascularization and vascular pathologies. Consequently, *Notch1*^+/−^; *Notch3*^−/−^ mice present with an accelerated CADASIL phenotype in developing retinal blood vessels and arteriovenous malformations.

## Results

### Notch is expressed by pericytes in the angiogenic retina

The role of Notch has been widely reported in the endothelium, where Notch1 and Notch4 regulate endothelial cell function^31^,^32^,^33^,^34^. Notch3 signaling regulates vascular smooth muscle cell differentiation, however less is known about the role of Notch receptors in pericytes^35^. Using a mouse reporter line for Notch activity we observed that pericytes lining retinal capillaries displayed evidence of active Notch signaling (Supplemental Fig. S1A). In vascular cells, we observed that Notch3 expression was restricted to mural cells, whereas endothelial cells and pericytes both expressed Notch1 (Supplemental Fig. S1B and C), indicating a possible cooperation of these receptors in mural cells during retinal angiogenesis. Cultured endothelial cells and pericytes expressed high levels of the Notch ligand JAGGED1, but did not express JAGGED2 (Supplemental Fig. SB). Expression of Jagged1 by both pericytes and endothelial cells in the developing retinal vasculature (Supplemental Fig. S1D) suggested that Jagged1 regulates Notch-dependent endothelial/pericyte cross-talk during angiogenesis.

### Notch1 and Notch3 cooperate to limit endothelial sprouting and promote vessel morphogenesis

To determine if Notch1 and Notch3 cooperate to regulate angiogenesis, we crossed *Notch3*^+/−^ mice to *Notch1*^+/−^; *Notch3*^+/−^ mice to obtain genotypes with varying levels of deficiency in Notch1 and Notch3 alleles; wildtype, *Notch3*^+/−^, *Notch3*^−/−^, *Notch1*^+/−^, *Notch1*^+/−^; *Notch3*^+/−^, and *Notch1*^+/−^; *Notch3*^−/−^. *Notch3*^−/−^ mice are viable, however *Notch1*^−/−^ mice die embryonically^36^,^37^. *Notch3*^+/−^ mice served as controls, as they were phenotypically indistinguishable from wildtype.

**Figure 1:**
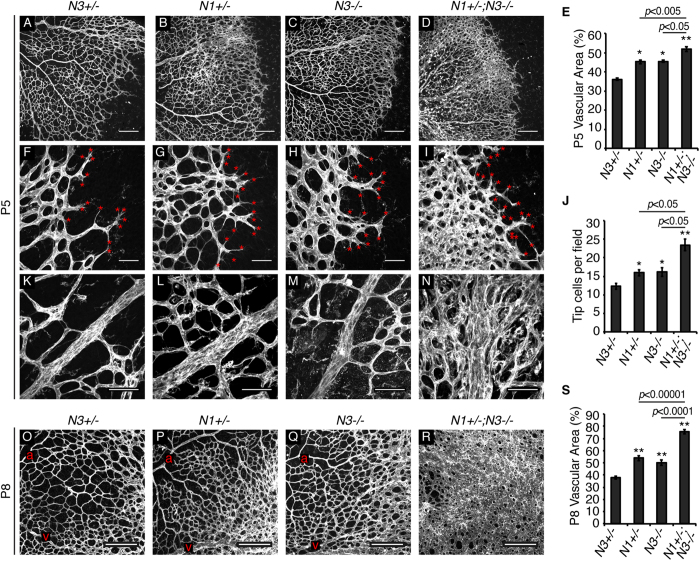
Notch1 and Notch3 limit vessel density during retinal angiogenesis. Retinas wholemount stained for isolectin B_4_ to visualize the vasculature. (**A**–**D**) Images of the retinal vascular plexus at P5. (**E**) Quantification of vessel density at P5. (**F**–**I**) Images of P5 retinas show tip cells (red asterisks) at the angiogenic front. (**J**) Quantification of average tip cell number per image field. (**K**–**N**) High magnification images of P5 venules immediately distal from the optic nerve. (**O**–**R**) P8 retinal vascular plexus (a = arteriole, v = venule). (**S**) Quantification of vessel density at P8. *N1* = *Notch1*, *N3* = *Notch3*. n≥3 mice per genotype, 2 retinas per mouse. Data are mean±s.e.m. *p < 0.01, **p < 0.001. Scale bars: (**A**–**D**,**O**–**R**) 200 μm, (**F**-**I**,**K**-**N**) 50 μm.

We assessed retinal vasculature of *Notch1*^+/−^, *Notch3*^−/−^, and *Notch1*^+/−^; *Notch3*^−/−^ mice at postnatal (P) developmental time points, P5 and P8. At P5, we observed increased vessel density in *Notch1*^+/−^ retinas, as compared to *Notch3*^+/−^ controls (Fig. 1A,B). This finding was expected, as Notch1 has been shown to function in endothelial cells to limit vessel sprouting^33^. Interestingly, *Notch3*^−/−^ mice also displayed a mild increase in vessel density over *Notch3*^+/−^ controls (Fig. 1C). Combined deficiency of Notch1 and Notch3 in P5 *Notch1*^+/−^; *Notch3*^−/−^ mice resulted in a significant increase in vessel density, as compared to *Notch1*^+/−^ and *Notch3*^−/−^ mice (Fig. 1D,E). We also observed a significantly higher number of endothelial tip cells at the angiogenic front of *Notch1*^+/−^; *Notch3*^−/−^ P5 retinas (Fig. 1I,J), suggesting that endothelial hypersprouting contributes to the observed increase in vessel density at P5. Enlarged venules were exclusively present in *Notch1*^+/−^; *Notch3*^−/−^ P5 retinas (Fig. 1N). Compared to *Notch3*^+/−^ controls, *Notch3*^−/−^ and *Notch1*^+/−^; *Notch3*^−/−^ mice displayed reduced vessel outgrowth at P5, (Supplemental Fig. S2).

At P8, *Notch1*^+/−^; *Notch3*^−/−^ mice maintained a significantly hyper-vascularized retinal primary plexus (Fig. 1R,S). *Notch1*^+/−^; *Notch3*^−/−^ P8 retinas displayed areas of sheet-like endothelium that lacked defined arterioles and venules, suggestive of impaired morphogenesis to a mature vascular plexus (Fig. 1R). Since Notch3 expression is restricted to mural cells, our data shows that the additional loss of Notch3 in mural cells of *Notch1*^+/−^; *Notch3*^−/−^ mice exacerbates the endothelial cell hyper-sprouting phenotype caused by Notch1 heterozygosity.

### Notch1 and Notch3 promote VSMC differentiation

We hypothesized that Notch1 and Notch3 may both be required for angiogenesis by ensuring normal mural cell function. To first determine if Notch1 and Notch3 function in VSMCs, we assessed P5 retinal arterioles. We found that *Notch3*^−/−^ mice displayed impaired arteriolar VSMC differentiation at P5, as evidenced by reduced αSMA expression (Supplemental Fig. S3), similar to what has been reported^26^. Additional deficiency of Notch1 in *Notch1*^+/−^; *Notch3*^−/−^ mice caused a further reduction in VSMC αSMA expression, suggesting that Notch1 and Notch3 cooperate to promote VSMC differentiation.

### Notch1 and Notch3 promote pericyte coverage of the retinal endothelium

**Figure 2:**
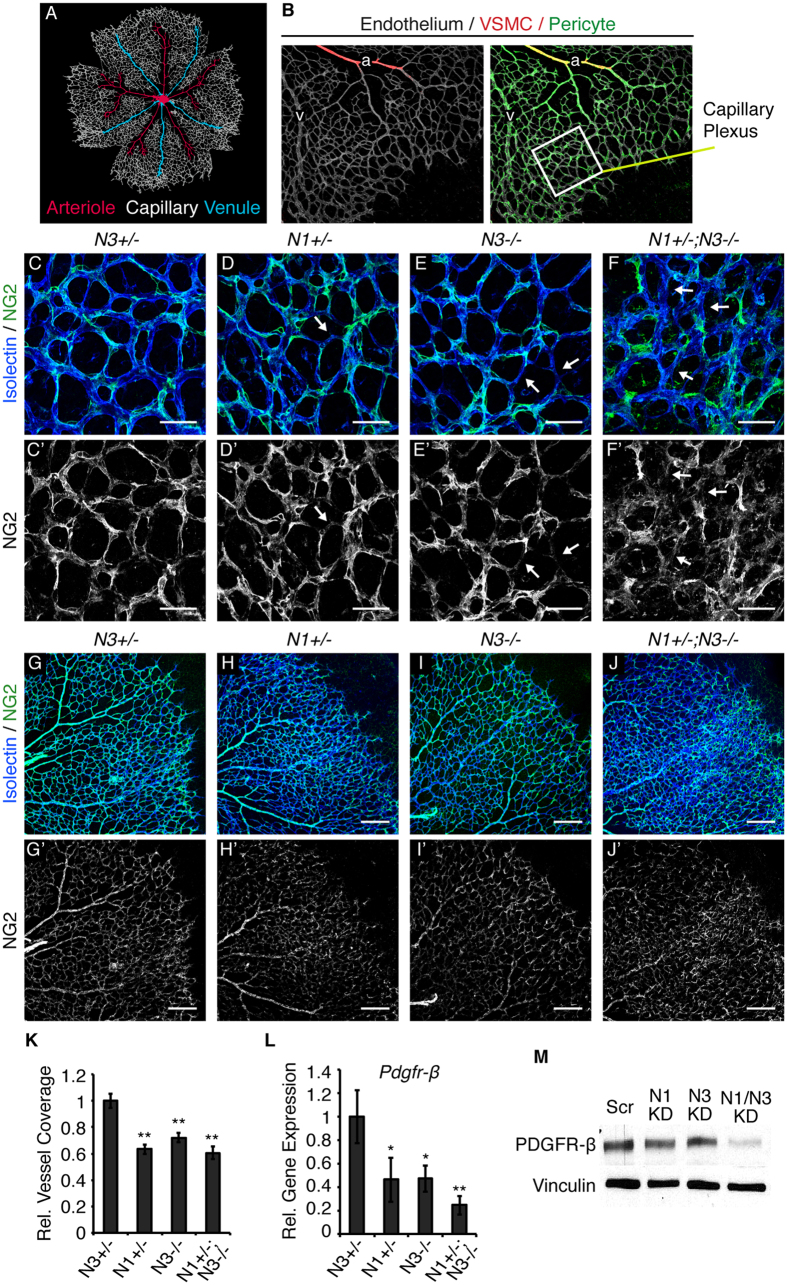
Notch deficiency impairs pericyte coverage. (**A**) Schematic of the vascular plexus of a P5 mouse retina with red arterioles, blue venules, and capillaries shown in grey. (**B**) P5 retina whole-mount stained for isolectin B_4_ (grey) to identify the endothelium. α-SMA (red) is restricted to VSMCs lining arterioles. NG2 (green) identifies pericytes on capillaries and venules and α-SMA-positive VSMCs. Area enclosed by white box represents an example of a region used for assessment of the capillary plexus. (**C**–**F**) P5 retinas whole-mount immunostained for isolectin B_4_ (blue) to mark endothelial cells and NG2 (green) to mark pericytes. (**C’**–**F’**) Corresponding NG2 staining in grey. White arrows mark endothelium devoid of pericyte coverage. (**G**–**J**) Example images used for quantification of pericyte content of P5 retinal vasculature stained for CD31 (blue) to mark endothelium and NG2 (green) to mark pericytes. (**G’**–**J’**) Corresponding NG2 staining in grey. (**K**) Quantification of vascular pericyte coverage by normalizing NG2 staining to CD31 in P5 retinas, relative to control. (**L**) *Pdgfr-β* transcript levels in P5 retinas measured by quantitative real time PCR, normalized to *gapdh* and relative to control. (**M**) Western blot for PDGFR-β and vinculin (loading control) on HBVP protein lysates. n≥3; *p < 0.01, **p < 0.0001. Data are mean±s.e.m. Scale bars: (**C**–**F’**) 25 μm, (**G**–**J’**) 200 μm.

Retinal mural cell populations can be identified as NG2-positive/αSMA-negative pericytes prevalent on capillaries and venules, and αSMA-positive VSMCs on arterioles (Fig. 2A,B). Given their localization to the capillary plexus and angiogenic front, pericytes, as compared to VSMCs, play a more active role in regulating sprouting angiogenesis. We hypothesized that pericyte/endothelial cell interactions were impaired in the hyper-vascularized retinas of *Notch1*^+/−^; *Notch3*^−/−^ mice. To visualize pericyte coverage we performed confocal imaging of P5 retinas wholemount stained for NG2 to mark pericytes and either CD31 or isolectin B_4_ to label the endothelium. The capillary endothelium of *Notch3*^+/−^ control mice was continuously lined by NG2-positive pericytes (Fig. 2C,C’). In contrast, gaps in pericyte coverage were observed in *Notch1*^+/−^; *Notch3*^−/−^ retinal capillaries (Fig. 2F,F’), as defined by sections of endothelium devoid of an overlaying NG2-postive signal. Gaps in pericyte coverage were also observed in *Notch1*^+/−^ and Notch3^−/−^ retinal capillaries, albeit to a lesser extent (Fig. 2D,E).

We quantified total pericyte content in the P5 retina and observed a significant reduction in pericyte number in *Notch3*^−/−^ retinas, as compared to control (Supplemental Fig. S4). Total pericyte content was unchanged in *Notch1*^+/−^ and *Notch1*^+/−^; *Notch3*^−/−^ retinas. To account for changes in vessel density, we normalized NG2 content relative to CD31-positive endothelial cells in low magnification images of P5 retinal vasculature (Fig. 2G–J’). Quantification of NG2 staining relative to vascular density showed reduced pericyte coverage in *Notch1*^+/−^*, Notch3*^−/−^, and *Notch1*^+/−^; *Notch3*^−/−^ P5 retinas, as compared to *Notch3*^+/−^ controls (Fig. 2K). As the presence of pericytes on growing vasculature was dependent on Notch function, this data points to a role for Notch in pericyte function during angiogenesis.

PDGFR-β signaling in pericytes drives their recruitment to new blood vessels. Given that reduced pericyte coverage was observed in mice with combined Notch1 and Notch3 deficiency, we analyzed *Pdgfr-β* expression by RT-PCR. We found that *Pdgfr-β* transcript levels were reduced in *Notch1*^+/−^*, Notch3*^−/−^, and *Notch1*^+/−^; *Notch3*^−/−^ P5 retinas (Fig. 2L). Additionally, knockdown of NOTCH1 (N1 KD) or NOTCH3 (N3 KD) in cultured human brain vascular pericytes (HBVP) reduced PDGFR-β protein expression, as compared to scrambled control (Scr) (Fig. 2M). Combined knockdown of NOTCH1 and NOTCH3 (N1/N3 KD) further reduced PDGFR-β protein levels (Fig. 2M, quantified in Supplemental Fig. S5A). Conversely, constitutive activation of Notch1 or Notch3 signaling induced *PDGFR-β* expression in cultured HBVP (Supplemental Fig. S5B). Taken together, these results demonstrate that Notch1 and Notch3 regulate pericyte PDGFR-β expression and that Notch promotes pericyte coverage during angiogenesis.

### Notch deficiency impairs pericyte association with the endothelium

**Figure 3:**
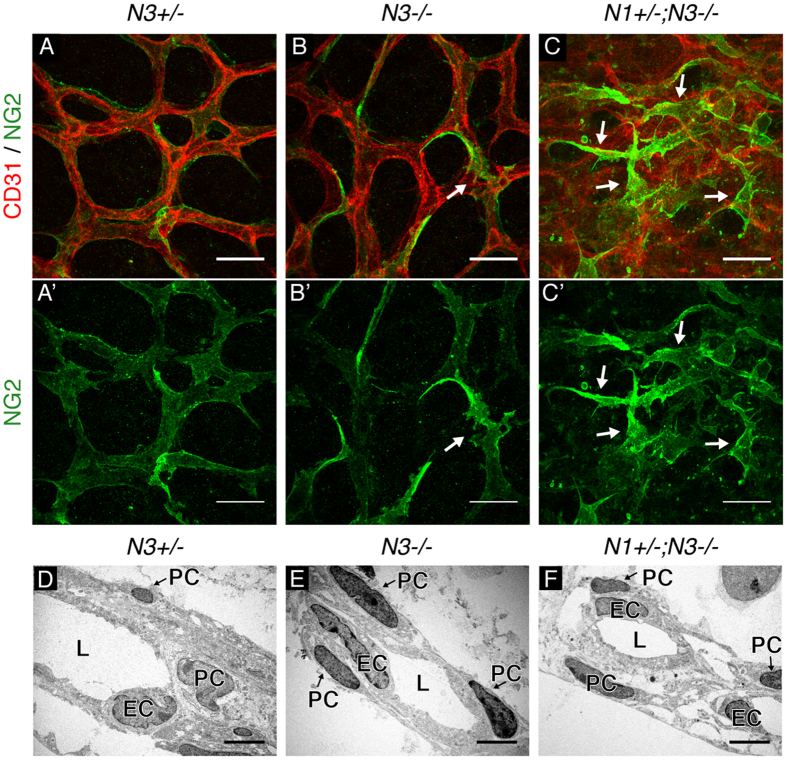
High magnification assessment of pericyte/endothelial cell interactions. (**A**–**C**) Capillary plexus of P5 retinas whole-mount immunostained for CD31 (red) to mark the endothelium and NG2 (green) to mark pericytes. (**A’**–**C’**) Corresponding NG2 staining in grey. Pericytes with abnormal cell morphology marked by white arrows. (**D**–**F**) Electron micrographs of retinal capillaries show vessel lumen (L), endothelial cell nuclei (EC), and pericyte nuclei (PC). Scale bars: (**A**–**C)** 25 μm, (**D**–**F**) 1 μm.

Analysis of *Notch1*^+/−^; *Notch3*^−/−^ retinal vasculature revealed heterogeneous NG2 staining intensity and irregular pericyte cell morphology. Thus, we hypothesized that combined deficiency of Notch1 and Notch3 may impair pericyte interaction with the endothelium. High magnification confocal images of P5 *Notch3*^+/−^ control retinas showed capillaries lined by NG2-positive pericytes that displayed a smooth cell surface and uniform NG2 staining (Fig. 3A,A’). In *Notch3*^−/−^ capillaries, a subset of pericytes displayed abnormally bright NG2 staining and altered cell morphology with abnormal cell processes (Fig. 3B,B’). *Notch1*^+/−^; *Notch3*^−/−^ pericytes displayed a more extreme phenotype with overlapping cell processes that failed to tightly and continuously line the endothelium (Fig. 3C,C’). We validated that the abnormal perivascular cells observed in *Notch1*^+/−^; *Notch3*^−/−^ capillaries were pericytes, as they co-expressed NG2 and an additional pericyte marker, desmin (Supplemental Fig. S6). We also observed reduced pericyte coverage and abnormal pericyte morphology along the enlarged retinal venules of *Notch1*^+/−^; *Notch3*^−/−^ P5 mice (Supplemental Fig. S7).

To gain a deeper understanding of the interaction between pericytes and the endothelium, we employed electron microscopy to visualize pericyte/endothelial cell association in the vessel wall of retinal capillaries. Electron micrographs of *Notch3*^+/−^ P5 mice showed a normal capillary vessel wall with pericytes tightly lining the blood vessels (Fig. 3D). Electron micrographs of *Notch3*^−/−^ P5 retinas revealed a normal capillary structure, indistinguishable from controls (Fig. 3E). *Notch1*^+/−^; *Notch3*^−/−^ mice displayed altered vessel structures with pericytes located at a greater distance from the vessel lumen, indicative of pericyte dissociation (Fig. 3F). Electron micrographs also suggested an altered vBM in *Notch1*^+/−^; *Notch3*^−/−^ retinal vessels where the intercellular region between pericytes and endothelial cells appeared wider, less dense and had visible large open spaces. Thus, confocal and electron microscopy analysis demonstrated that *Notch1*^+/−^; *Notch3*^−/−^ retinal vessels are lined by pericytes that fail to associate properly with the endothelium.

### Notch1 and Notch3 cooperate in vascular basement membrane formation

**Figure 4:**
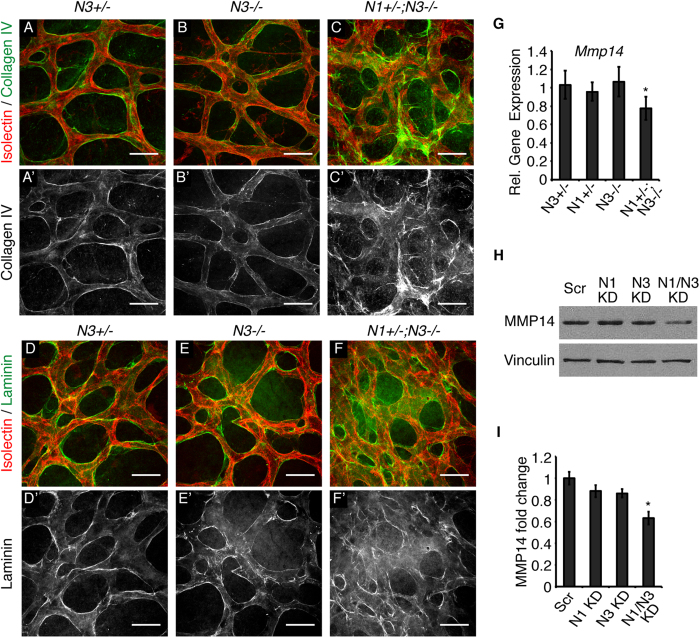
Notch1 and Notch3 cooperate to regulate the vascular basement membrane. (**A**–**C**) High magnification images taken at the capillary plexus of P5 retinas whole-mount immunostained with isolectin B_4_ (red) and collagen IV (green). (**A’**–**C’**) Corresponding collagen IV staining in grey. (**D**–**F**) P5 retinal capillary plexus stained with isolectin B_4_ (red) and laminin (green). (**D’**–**F’**) Corresponding laminin staining in grey. (**G**) *Mmp14* transcript levels in P5 retinas measured by quantitative real time PCR, normalized to *gapdh* and relative to control. (**H**) Western blot for MMP14 and vinculin (loading control) on HBVP protein lysates. (**I**) MMP14 protein levels in HBVP cell lysates, normalized to vinculin and relative to control. n≥3; *P < 0.05, **P < 0.0001. Data are mean±s.e.m. Scale bars: 25 μm.

To further investigate the abnormal vBM suggested by electron micrographs of P5 *Notch1*^+/−^; *Notch3*^−/−^ retinas, we assessed two basement membrane components, collagen IV and laminin by immunohistochemistry. *Notch3*^+/−^ control mice and *Notch3*^−/−^ mice displayed a uniform layer of the vBM component collagen IV lining the capillary endothelium (Fig. 4A,B). Normal Collagen IV staining was observed in the retinal capillaries of *Notch1*^+/−^ mice (Supplemental Fig. S8). In *Notch1*^+/−^; *Notch3*^−/−^ capillaries, collagen IV deposition was severely disorganized and prevalently deposited in the open spaces of the capillary plexus (Fig. 4C,C’). Abnormal laminin deposition was also observed in *Notch1*^+/−^; *Notch3*^−/−^ capillaries (Fig. 4F,F’). *Notch3*^−/−^ retinas displayed normal collagen IV and laminin deposition along retinal capillaries (Fig. 4B,E). We observed abnormal vBM deposition only in *Notch1*^+/−^; *Notch3*^−/−^ retinas, suggesting that Notch1 and Notch3 cooperate to regulate the vBM.

Matrix metalloproteinase (MMP)14 and its co-activator MMP2 function in vBM homeostasis to locally degrade vBM components, such as collagen IV, to form an organized and effective vBM^38^. Accordingly, reduced MMP expression and/or activity can cause vBM abnormalities. Notch signaling induces MMP14 expression and regulates MMP2 activity in cultured endothelial cells^39^. Thus, we investigated whether MMP14 was altered with combined deficiency of Notch1 and Notch3 *in vivo*. Results showed reduced *Mmp14* transcript levels in RNA isolated from *Notch1*^+/−^; *Notch3*^−/−^ P5 retinas (Fig. 4G) when compared to *Notch3*^+/−^*, Notch1*^+/−^, or *Notch3*^−/−^ retinas. Additionally, reduced protein levels of MMP14 and MMP2 were observed in cultured pericytes only when both NOTCH1 and NOTCH3 expression was reduced (Fig. 4H,I, Supplemental Fig. S9). Taken together, these findings demonstrate that Notch1 and Notch3 cooperate to regulate MMP2 and MMP14 expression in pericytes and suggest that both Notch1 and Notch3 are required for proper MMP expression to ensure vBM formation during retinal angiogenesis.

### *Notch1* ^+/−^; *Notch3* ^−/−^ mice display a CADASIL phenotype in developing vasculature

**Figure 5:**
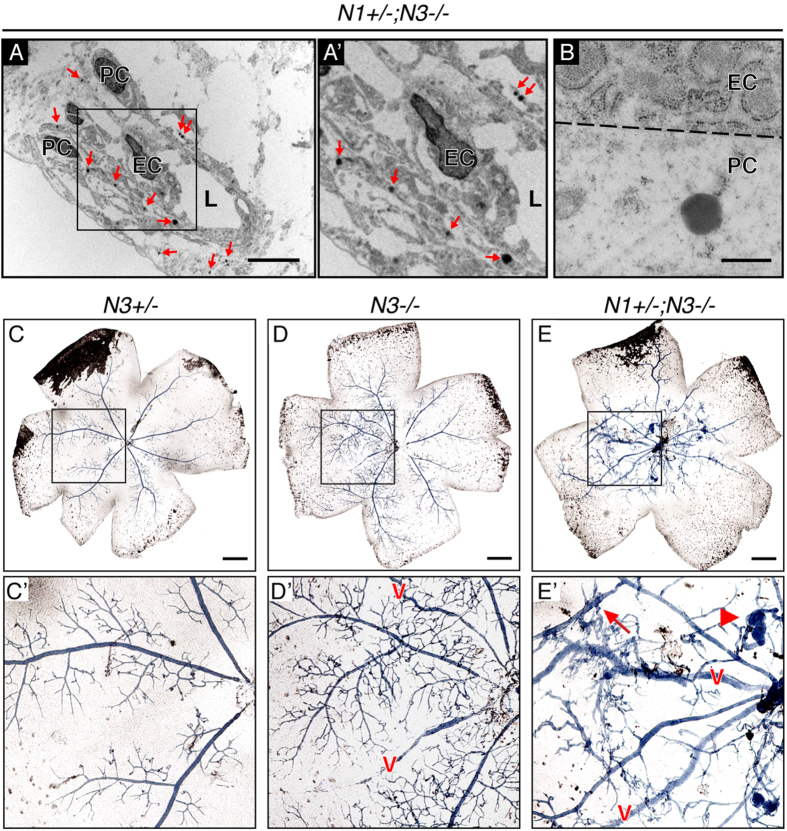
Assessment of vascular pathology in *Notch1*^+/−^; *Notch3*^−/−^ retinas. (**A**) GOM deposits (red arrows) associated with the mural cell compartment in electron micrographs of P5 *Notch1*^+/−^; *Notch3*^−/−^ retinal vessels modeling CADASIL. (**A’**) Area enclosed by black box in **A**. (**B**) 50,000× magnification of a GOM associated with a pericyte, endothelial plasma membrane represented by dashed line. Vessel lumen (L), endothelial cell nuclei (EC), and pericyte nuclei (PC). (**C**–**E**) Bright field images of whole-mount P13 retinas following blue liquid latex perfusion to assess for AVMs. (**C’**–**E’**) Zoomed image of area enclosed by black square in upper panel. Blue latex perfused venules marked with a red v. *Notch1*^+/−^; *Notch3*^−/−^ mice (**E’**) display vascular tangles (arrowhead) and arteriovenous shunts (arrow). Scale bars: (**A**) 1 μm, (**B**) 0.5 μm, (**C**–**E**) 500 μm.

The retinal vessels of P5 *Notch1*^+/−^; *Notch3*^−/−^ mice displayed loss of mural cells, one of the hallmarks of the NOTCH3-linked small artery disease CADASIL. CADASIL is caused by mutations in NOTCH3 and is characterized by a gradual loss of arterial mural cells and accumulation of dark deposits of granular osmiophilic material (GOM) in the basal lamina of pericytes and VSMCs^7^. Further examination of electron micrographs of P5 retinal capillaries of *Notch1*^+/−^; *Notch3*^−/−^ mice showed numerous GOM deposits, mirroring CADASIL pathologies (Fig. 5A,A’). GOMs were always observed in the vicinity of pericytes and never associated with endothelial cells (Fig. 5B), highlighting pericyte dysfunction. GOM deposits were not observed in *Notch3*^+/−^ or *Notch3*^−/−^ mice (Fig. 3D,E); in line with previous reports that *Notch3*^−/−^ mice do not recapitulate a CADASIL phenotype^25^,^27^. These findings demonstrate that combined deficiency of Notch1 and Notch3 models hallmarks of CADASIL pathology in the developing retina and further support a role for Notch signaling in pericyte function.

### *Notch1* ^+/−^; *Notch3* ^−/−^ mice display retinal arteriovenous malformations

The pericyte dysfunction phenotype, angiogenic defects, and altered vBM observed in *Notch1*^+/−^; *Notch3*^−/−^ retinas suggested that vascular function may be impaired in these mutant mice. In P13 mice, the retinal vascular plexus has sufficiently matured into arterioles, venules and intervening capillaries to allow for the segregation of oxygenated and deoxygenated blood^6^,^40^. Arteriovenous malformations (AVMs) occur when abnormal arteries and veins make direct connections through either a shunt or an abnormal intervening capillary plexus^41^. We assessed P13 retinas for AVMs using a liquid latex perfusion assay, where blue liquid latex, which is excluded from the small lumen size of normal capillaries, is injected into arterial circulation^42^. In normal vasculature of *Notch3*^+/−^ mice, liquid latex delivery was limited to the arterial side of circulation (Fig. 5C,C’). Retinas from *Notch3*^−/−^ mice presented with at least one blue venule, where abnormal capillary connections filled with blue latex can be seen feeding the venules (Fig. 5D,D’). A severe AVM phenotype was observed in *Notch1*^+/−^; *Notch3*^−/−^ mice; all retinas were positive for blue venules (Fig. 5E). Moreover, *Notch1*^+/−^; *Notch3*^−/−^ retinas showed extensive presence of arteriovenous shunts and vascular tangles (Fig. 5E’). Thus, additional deficiency of Notch1 in a Notch3 null background caused extensive retinal AVMs with vascular tangles resembling characteristic human AVM *nidus*. This is the first evidence that Notch3 deficiency promotes vascular shunts and contributes to AVM formation.

## Discussion

Here we demonstrate a novel role for mammalian Notch in angiogenesis. We show that Notch signaling regulates pericyte coverage and pericyte-endothelial cell interactions to promote vessel quiescence and maturation. We observed pericyte and endothelial cell dysfunction due to combined Notch deficiency, where loss of mural cell Notch3 exacerbates the endothelial hyper-sprouting caused by Notch1 heterozygosity. Notch3 deletion combined with Notch1 haploinsufficiency also resulted in a CADASIL-like phenotype in developing retinal vessels. Furthermore, *Notch1*^+/−^; *Notch3*^−/−^ mice displayed retinal AVMs, pointing to a key role for pericytes in normal vascular function and indicating that pericyte dysfunction due to Notch deficiency contributes to arteriovenous malformations.

We observed reduced pericyte coverage of the retinal vasculature of *Notch3*^−/−^ mice. Our findings support previous reports on Notch3 in zebrafish, where Notch3 deficiency caused reduced pericyte coverage in the brain^28^,^43^. Our data is also in line with previously reported *in vitro* work showing that pericyte Notch3 is required for pericyte adhesion to endothelial cells^30^. Previous publications have reported impaired VSMC function, yet normal pericyte coverage in mice mutant for Notch3^26^,^27^. Our findings may differ from those previously reported because we employed different mutant Notch3 mouse lines and assessed pericyte coverage during development, as opposed to adulthood.

Our work expands upon the role of Notch in angiogenesis by demonstrating that Notch signaling in pericytes can function to limit endothelial cell sprouting. Notch signaling has been studied in depth in the endothelium, where Notch1 activation through Dll4 regulates angiogenesis by inhibiting endothelial cell sprouting^21^,^22^,^33^. We observed that *Notch3*^−/−^ mice displayed increased vessel density and an increased number of endothelial tip cells during angiogenesis in the retina. In retinal capillaries, Notch3 expression is restricted to pericytes. We can thus likely attribute these aforementioned *in vivo* vascular defects to Notch deficiency in pericytes and therefore demonstrate that pericyte dysfunction contributes to vascular phenotypes during angiogenesis. Jagged1 has been shown to modulate Notch signaling in both endothelial cells and VSMCs^44^,^45^. We showed that both endothelial cells and pericytes express Jagged1 during retinal angiogenesis. We propose that Jagged1 expression by either of these cell types can function to regulate Notch signaling and thus promote pericyte/endothelial and pericyte/pericyte interactions critical for angiogenesis.

*Notch1*^+/−^; *Notch3*^−/−^ mice displayed increased vessel density at P5, as compared to *Notch1*^+/−^ or *Notch3*^−/−^ mice. We believe that combined deficiency of Notch1 and Notch3 contributes to this dramatic vessel density phenotype in two ways. Firstly, we propose that pericyte function is more severely impaired when both Notch1 and Notch3 signaling is reduced. Secondly, Notch1 deficiency in the endothelium causes endothelial hyperactivation due to increased VEGFR-2 expression^46^. It is reasonable to assume that endothelial hyperactivation is compounded by pericyte dysfunction resulting in impaired endothelial quiescence and vessel maturation. Future studies on pericyte-specific deletion of Notch will contribute to our understanding of the cell autonomous role for Notch in pericyte function. Nonetheless, the findings reported here shed light on the cellular and molecular mechanisms that govern pathological angiogenesis characterized by uncontrolled and excessive vascularization. Promotion of proper pericyte coverage could have therapeutic potential to limit pathological angiogenesis by inducing vessel quiescence.

Pericytes share a basement membrane with the endothelium and pericyte recruitment to nascent blood vessels is critical for proper vBM deposition^12^,^47^. Previous studies in the mouse retina demonstrated that vBM can limit endothelial cell sprouting by inducing endothelial Notch signaling^48^,^49^. Our results show that *Notch1*^+/−^; *Notch3*^−/−^ mice displayed severe disorganization of two vBM components, collagen IV and laminin, in the capillary plexus of the retina. We also observed that, as compared to *Notch1*^+/−^ mice, *Notch1*^+/−^; *Notch3*^−/−^ mice displayed increased vessel density and an increased number of tip cells during angiogenesis. Hence, in addition to pericyte dysfunction, alterations in vBM in *Notch1*^+/−^; *Notch3*^−/−^ mice may also contribute to a worsened hyper-sprouting phenotype by further reducing endothelial Notch signaling.

Our data suggests that altered MMP expression in *Notch1*^+/−^; *Notch3*^−/−^ mice contributed to vascular basement membrane alterations. MMP2 is activated by MMP14 to degrade specific components of extracellular matrices, including collagen IV, and we previously established that Notch signaling promotes MMP14 expression and MMP2 activity in endothelial cells^39^. Here we showed that Notch regulates MMP14 expression in cultured pericytes and in retinas of neonatal mice, and that collagen IV is disorganized in *Notch1*^+/−^; *Notch3*^−/−^ retinal vasculature. Based on these findings, we propose that Notch regulation of MMP14 expression in pericytes and endothelial cells functions to regulate proper basement membrane deposition. Lack of adequate MMP activity impedes basement membrane formation, likely contributing to the pericyte dissociation phenotype observed in *Notch1*^+/−^; *Notch3*^−/−^ retinas.

We observed reduced pericyte coverage of *Notch1*^+/−^, *Notch3*^−/−^ and *Notch1*^+/−^; *Notch3*^−/−^ retinal vasculature. Adequate pericyte recruitment and survival, and proper pericyte interaction with the endothelium ensures coverage of microvessels with pericytes. Impairment of any of these three processes could result in reduced pericyte coverage during angiogenesis. Our data demonstrates that both Notch1 and Notch3 promote pericyte PDGFR-β expression, the major tyrosine kinase responsible for pericyte recruitment. Deficient PDGFR-β signaling may contribute to the reduced pericyte coverage observed in mice mutant for Notch1 and Notch3 by inhibiting pericyte recruitment to nascent blood vessels. Notch signaling has also been show to promote retinal pericyte survival, thus providing a second possible explanation for reduced pericyte coverage in Notch mutant mice^29^. Lastly, pericytes may fail to associate properly with the endothelium due to an impaired ability for pericytes to adhere to the vBM in *Notch1*^+/−^; *Notch3*^−/−^ vasculature. Vascular cells utilize a multitude of trans-membrane integrin receptors to interact with the basement membrane^50^. Notch signaling in retinal αSMA-positive VSMCs has been shown to regulate integrin αvβ3 expression allowing for adhesion of VSMCs to the basement membrane^51^. Vascular integrin expression may be altered in *Notch1*^+/−^; *Notch3*^−/−^ mice, thus impairing pericyte adhesion to basement membrane and contributing to reduced pericyte coverage. A better understanding of how Notch governs the processes that regulate pericyte coverage could expand our therapeutic potential in treating diseases characterized by mural cell dysfunction.

Our data demonstrates a dual contribution of Notch1 and Notch3 to mural cell function during developmental angiogenesis. Thus, in our mouse model Notch1 haploinsufficiency, combined with Notch3 nullizygosity further weakens mural cells, and their ability to associate with the endothelium. We propose that impaired mural cell function due to combined deficiency of Notch1 and Notch3 caused an accelerated CADASIL phenotype characterized by mural cell dissociation and the appearance of GOMs. This is supported by the fact that GOMs were only observed in *Notch1*^+/−^; *Notch3*^−/−^ and not *Notch3*^−/−^ retinal vessels. NOTCH1 may compensate for impaired NOTCH3 function in CADASIL patients, thus combined deficiency in NOTCH1 and NOTCH3 would cause an accelerated CADASIL phenotype.

Observation of a CADASIL phenotype in neonatal developing retinal vessels was a novel finding. Transgenic expression of characterized mutations of NOTCH3 have been shown to cause a CADASIL phenotype only in adult mice; evaluation of younger mice revealed normal vasculature^52^. CADASIL-like lesions in infants and children have yet to be reported, however, early stage lesions would be difficult to detect. Our work implies that there may be early onset CADASIL cases associated with more severe NOTCH deficiencies.

A noteworthy aspect of the Notch loss of function phenotypes in *Notch1*^+/−^; *Notch3*^−/−^ mice is the appearance of GOMs. Extracellular aggregates of the NOTCH3 extracellular domain (NOTCH3_ECD_) have been documented in CADASIL mouse models and human patients^53^,^54^. It is hypothesized that NOTCH3_ECD_ aggregates contribute to disease progression. Our observation of CADASIL pathology in complete absence of Notch3 suggests that NOTCH3_ECD_ aggregates are a consequence of CADASIL and not causative.

AVM etiology and development remains largely unknown, particularly in the retina. Hallmarks of AVMs are considered high flow lesions in which arteries and veins are connected, bypassing the capillary network, and creating entangled and tortuous vessels (*nidus*). We have found that *Notch1*^+/−^; *Notch3*^−/−^ mutant mice present enlarged and tortuous connections directly between arteries and veins bypassing capillaries. Due to the difficulties of applying such a technique in small animals, we have not had the opportunity to verify high flow in such lesions, but most of the hallmarks analyzed point to the development of *bona fide* AVMs in *Notch1*^+/−^; *Notch3*^−/−^ mutant mice. Future work will focus on further evaluation of AVMs in other organs, as well as in aged mice.

We propose that the AVMs and CADASIL pathology observed in *Notch1*^+/−^; *Notch3*^−/−^ mice constitute two separate pathological events. Our data suggests that the CADASIL phenotype originates from mural cell dysfunction because of enhanced deterioration when these cells lack both Notch1 and Notch3. In contrast, the development of AVMs likely arises from a combination of endothelial dysfunction due to Notch1 heterozygosity, in addition to mural cell dysfunction caused by combined deficiency of Notch1 and Notch3. This proposal is based upon the fact that endothelial dysfunction has been implicated in the development of AVMs. However, the pathogenesis of AVMs remains unclear and a mural cell contribution to AVMs has been largely overlooked. Decreased mural cell coverage has been seen in a subset of human AVMs, as well as in mouse models^55^,^56^. Endothelial Notch signaling has been previously associated with AVMs; genetic mouse models with either gain of function or loss of function of Notch develop AVMs^57^,^58^,^59^,^60^. Here we provide the first evidence for a role for Notch in the mural cell compartment during the formation of AVMs. Our data showed that loss of Notch3 expression in mural cells contributes to AVMs, thereby expanding the functional role of Notch in these vascular abnormalities. In conclusion, we propose a model where pericyte dysfunction, due to combined deficiency of Notch1 and Notch3, combined with endothelial activation, due to Notch1 haploinsufficiency, results in AVMs. Analysis of AVMs in mouse models with tissue-specific deletion of Notch could help address this hypothesis.

The severity of the vascular phenotypes displayed by *Notch1*^+/−^; *Notch3*^−/−^ mice highlights an important role for Notch signaling in pericyte function. Pericyte dysfunction has been reported in vasculopathies such as diabetic retinopathy and stroke. We found that altered pericyte function due to Notch deficiency disrupts retinal angiogenesis and contributes to arteriovenous malformations. Our study of Notch deficient mice suggests that the role of Notch in pericyte/endothelial cell interactions is a previously unappreciated and critical component of vascular disease.

## Materials and Methods

### Mice

*Notch1* and *Notch3* mutant mice were previously described and maintained in a C57BL/6 background^36^,^37^. All procedures were performed in accordance with approved protocols and guidelines established by the Columbia University Institutional Animal Care and Use Committee.

### Statistical Analysis

A two-tailed, unpaired Student’s t test was used to assess statistical significance (Microsoft Excel).

### Retinal Whole-mount Immunohistochemistry

Following sacrifice, eyes were enucleated and fixed in 4% paraformaldehyde for 2 hours at 4°C. Retinas were dissected from the eye and incubated in blocking solution (0.5% Triton–X-100, 1% BSA in PBS) for 3 hours at room temperature. Retinas were stained with the following primary antibodies at 4°C overnight in PBLEC (1% Triton-X-100, 1 mM MgCl2, 1mM MnCl2, and 1mM CaCl2 in PBS [pH 6.8]); Biotinylated isolectin B_4_ (1:50; Vector), anti-CD31 (1:100, BD Pharmingen), anti-NG2 (1:500, Millipore), anti-desmin (1:25, R&D), anti-αSMA-Cy3 (1:750, Sigma), anti-Notch3 (1:200, Santa Cruz), anti-collagen type IV (1:500, Cosmo. Bio), anti-laminin (1:500, Abcam), and 488-conjugated anti-GFP (1:200, Invitrogen). After washing with PBLEC, retinas were incubated with Alexa Fluor conjugated secondary antibodies (1:500, Invitrogen) in PBLEC. Stained retinas were flat mounted in 90% glycerol and confocal stacked images were acquired using a Nikon A1R microscope. Quantification of vessel density and pericyte coverage was performed using Image J on four 10× confocal stacked images per retina. Tip cells were defined as filopodia bursts and counted manually on four 40× images per retina.

### Retinal RNA Extraction

To obtain retinal RNA, eyes were enucleated following sacrifice and immediately transferred to RNA-later (Invitrogen) and placed on ice. Eyes remained in RNA later while retina tissue was dissected from the eye and then homogenized using a tissue homogenizer (Omni International) in RNeasy cell lysis buffer (Qiagen). RNA was collected using the RNeasy Mini Kit (Qiagen). Procedures for reverse transcription to cDNA, and quantitative real-time PCR, as well as primers are described in supplemental information.

### Cell culture and Analysis

Human brain vascular pericytes (HBVP) (ScienCell) were maintained in 1× Low Glucose DMEM (Invitrogen) with 10% fetal bovine serum and 1× Pen-Strep (Invitrogen). Human umbilical vein endothelial cells (HUVEC) were isolated as described^61^. HUVEC were grown in EGM-2 Endothelial Cell Growth Media (Lonza Group). Knockdown cell lines were made using stable lentiviral infection (see supplemental information). RNA was collected from cultured cells using the RNeasy Mini Kit (Qiagen). Protein lysates was collected 4 days post-infection in TENT lysis buffer containing 1× HALT protease inhibitor cocktail (Thermo Fisher). The following antibodies were used for western blotting in 2% milk, 2% BSA, 0.1% Tween in 1× PBS: anti-NG2 (Millipore), anti-PDGFR-β (Cell Signaling), anti-MMP14 (Abcam), anti-MMP2 (Abcam), anti-vinculin (Sigma), and HRP-conjugated secondary antibodies (Sigma).

### Transmission Electron Microscopy

Eyes were enucleated and fixed in 4% PFA in 0.1 M Sorenson’s buffer (PH 7.2) for 3 hours at 4°C. Retinas were dissected from the eye and further fixed with 2.5% gluteraldehyde and then post-fixed with 1% OsO4, both in Sorenson’s buffer, for 1 hour. Tissue was embedded in Lx-112 (Ladd Research Industries, Inc.). 60 nm sections were cut on a PT-XL ultramicrotome and then stained with uranyl acetate and lead citrate. Images were captured using a JEOL JEM-1200 EXII electron microscope with an ORCA-HR digital camera (Hamamatsu).

### Blue Liquid Latex Perfusion Assay

Liquid latex perfusion was performed as described^42^. Briefly, a lethal dose of anesthesia (0.5 mg/g of ketamine/0.1 mg/g of xylazine) was administered and blue liquid latex (Connecticut Valley Biological Supply Company) was injected into the left ventricle of the heart prior to sacrifice. Eyes were then enucleated and processed for retinal whole-mount. Brightfield 10× tiled images were obtained using a PALM MicroBeam IV Laser Capture, Microirradiation, Histology Inverted microscope (Zeiss).

## Additional Information

**How to cite this article**: Kofler, N. M. *et al.* Combined deficiency of Notch1 and Notch3 causes pericyte dysfunction, models CADASIL, and results in arteriovenous malformations. *Sci. Rep.* **5**, 16449; doi: 10.1038/srep16449 (2015).

## Supplementary Material

Supplementary Information
