# Supplementary material for: Combined deficiency of Notch1 and Notch3 causes pericyte dysfunction, models CADASIL, and results in arteriovenous malformations
Source: Sci Rep. 2015 Nov 13;5:16449. doi: 10.1038/srep16449 (PMC4643246; doi:10.1038/srep16449)
Supplement: Supplementary Information [file srep16449-s1.pdf]

Combined deficiency of Notch1 and Notch3 causes pericyte dysfunction, models CADASIL, and results in arteriovenous malformations

Natalie M. Kofler[1], Henar Cuervo[1], Minji K. Uh[1], Aino Murtomäki[1,2], and Jan Kitajewski[1,3,4]

Supplemental Information

Supplemental Materials and Methods

Production of cDNA and assessment by quantitative real-time PCR

Isolated RNA was treated with DNase1 (Invitrogen) for 30 minutes and then used in reverse-transcription PCR using the Verso™ Reverse Transcriptase kit with random hexamers to prime cDNA (Thermo Fisher). For quantitative RT-PCR, the reactions were done in triplicate with ABsolute Blue QPCR SYBR Green ROX Mix (Thermo Scientific) using a 7300 Real-Time PCR System (Applied Biosystems). Standard PCR to assess relative gene expression levels were performed by amplifying cDNA using specific primers and Platinum Taq Polymerase (Invitrogen). PCR amplification was performed with 35 cycles. The following human primer pairs were used: *NOTCH1* (5'-CTCACCTGGTGCAGACCCAG-3', 5'-GCACCTGTAGCTGGTGGCTG-3'), *NOTCH3* (5'-CGCCTGAGAATGATCACTGCTTC-3', 5'-TCACCCTTGGCCATGTTCTTC-3'), JAGGED1 (5'- GCTTGGATCTGTTGCTTGGTGAC-3', 5-ACTTTCCAAGTCTCTGTTGTCCTG-3'), JAGGED2 (5'- GCTATTTCGAGCTGCAGCTGAG-3', 5'- GCGGCAGGTAGAAGGAGTTG-3'), *HEYL* (5'- CAGGATTCTTTGATGCCCGAG-3', 5'-GACAGGGCTGGGCACTCTTC-3'), *PDGFR-β* (5'- ATGCCTCCGACGAGATCTATG-3', 5'-TTGAGGAGGTGTTGACTCATTC-3'), and *β-ACTIN* (5'- CGAGGCCCAGAGCAAGAGAG-3', 5'-CTCGTGATGGGCACAGTGTG-3'). The following mouse primer pairs were used: *pdgfr-β* (5'-ATGCCTCCGACGAGATCTATG-3', 5'-TTGAGGAGGTGTTGACTCATTC-3'), *mmp14* (5'-CAGTATGGCTACCTACCTCCAG-3', 5'-GCCTTGCCTGTCACTTGTAAA-3'), *gapdh* (5'-AACTTTGGCATTGTGGAAGG-3', 5'-ACACATTGGGGGTAGGAACA-3'). All experiments were

performed in triplicate. A two-tailed, unpaired Student's t test was used to assess statistical significance (Microsoft Excel).

Production of stable cell lines

Lentivirus was produced by 293T cells that were calcium phosphate transfected with viral packaging vectors and appropriate lentiviral constructs. For Notch activation, 293T cells were transfected with 10 µg of pccl-GFP, pccl-N1IC, or pcclN3IC. For Notch knockdown experiments 293T were transected with 5 µg plko-empty vector plus 5 µg plko-scrambled shRNA, or 5 µg plko-NOTCH1 shRNA, or 5 µg plko-NOTCH3 shRNA. For double knockdown cells were transfected with 5 µg plko-NOTCH1 shRNA and 5 µg plko-NOTCH3 shRNA. To make stable cell lines, 293T supernatant containing virus was passed through a 0.45 µm filter and added to target HBVP cells. All knockdown constructs were obtained from Sigma and validated by quantitative real-time PCR.

Supplemental Figure Legends

Supplemental Fig. S1. Notch1 and Notch3 are expressed by pericytes and Notch3 expression is restricted to mural cells in the P5 retina (A) The vascular plexus of P5 retinas isolated from Transgenic Notch Reporter (TNR) mice stained for GFP (green) to visualize Notch activation in isolectin B_4-positive endothelial cells (blue) and NG2-positive pericytes (red). Area enclosed by white box shown in lower panel (B) PCR amplification using primers specific for *NOTCH1* and *NOTCH3* of cDNA isolated from human umbilical venous endothelial cells (HUVEC) or HBVP with *β-ACTIN* as control. (C) P5 retinas stained for isolectin B_4 (blue) to mark endothelial cells, NG2 (red) to mark pericytes and Notch3 (green). Area enclosed by white box shown in lower panel. Scale bars: (A) 25 µM, (C) 50 µM.

Supplemental Fig. S2. *Notch1$^{+/-}$;Notch3$^{-/-}$* mice display reduced retinal vessel outgrowth. P5 retinas wholemount stained for isolectin B_4 to visualize the vasculature. Graph shows quantification of vessel outgrowth. Vessel outgrowth was assessed by measuring the distance between the optic nerve and angiogenic front of each retinal leaflet on 4x images using Image J. n≥3; *P<0.01, **P<0.001. Data are mean±s.e.m. Scale bars: 500 μm.

Supplemental Fig. S3. Assessment of VSMC maturation in retinas deficient for Notch1 and Notch3. Images of P5 arterioles taken at the first branch point distal to the optic nerve in retinas whole-mount immunostained for CD31 (blue) to identify endothelial cells, αSMA (red) to identify VSMCs, and NG2 (green) to identify mural cells. Scale bars: 50 μm.

Supplemental Fig. S4. Quantification of total retinal pericyte content of P5 retinas. Total pericyte content determined by measuring the percentage of retinal area positive for NG2 staining. Quantification performed on retinas assessed in Figure 2K. n≥3; *p<0.01. Data are mean±s.e.m.

Supplemental Fig. S5. Notch regulates *PDGFR-β* in cultured pericytes. (A) Quantification of western blot show in Figure 2M of results section: PDGFR-β protein levels normalized to vinculin and relative to scramble RNA (Scr) control in HBVP expressing NOTCH1 shRNA (N1 KD), NOTCH3 shRNA (N3 KD) or both NOTCH1 and NOTCH3 shRNA (N1/N3 KD). (B) Quantitative real time PCR of *PDGFR-β* gene expression, normalized to *GAPDH* in HBVP cell lines expressing activated NOTCH1 (N1IC), activated NOTCH3 (N3IC) or GFP as control. Increased levels of the downstream Notch target gene *HEYL* validated Notch activation. All quantification based on three independent experiments. *P<0.05, **P <0.01. Data are mean±s.e.m.

Supplemental Fig. S6. Validation of pericyte identity in *Notch1$^{+/-}$;Notch3$^{-/-}$* retinal capillaries. High magnification images of the capillary plexus of P5 mice show perivascular cells co-expressing pericyte markers NG2 (green) and desmin (blue). Isolectin (red) marks the endothelium. Scale bars: 50 μM.

Supplemental Fig. S7. Reduced pericyte coverage of P5 *Notch1$^{+/-}$;Notch3$^{-/-}$* venules. High magnification images of venules taken at the first branch point from the optic nerve of retinas wholemount stained for CD31 (red) to mark endothelial cells and NG2 (green) to mark pericytes. Quantification of pericyte coverage normalized to venule area. n≥3; *P<0.05, **P<0.001. Data are mean±s.e.m. Scale bars: 25 μm.

Supplemental Fig. S8. Evaluation of Collagen IV expression in *Notch1$^{+/-}$* P5 retina. (A-C) High magnification images taken at the capillary plexus of P5 retinas whole-mount immunostained with isolectin B_4 (blue) and collagen IV (green). (A'-C') Corresponding collagen IV staining in grey. Scale bars: 50 μM.

Supplemental Fig. S9. Notch regulates MMP2 expression in cultured pericytes. Western blot probed for MMP2 and vinculin (loading control) on protein lysates from HBVP expressing scrambled shRNA as control (Scr) or shRNA against NOTCH1 (N1 KD), NOTCH3 (N3 KD), or both NOTCH1 and NOTCH3 (N1/N3 KD). Quantification of MMP2 protein levels normalized to vinculin and relative to control, and carried out on three independent experiments. *P<0.05. Data are mean±s.e.m.

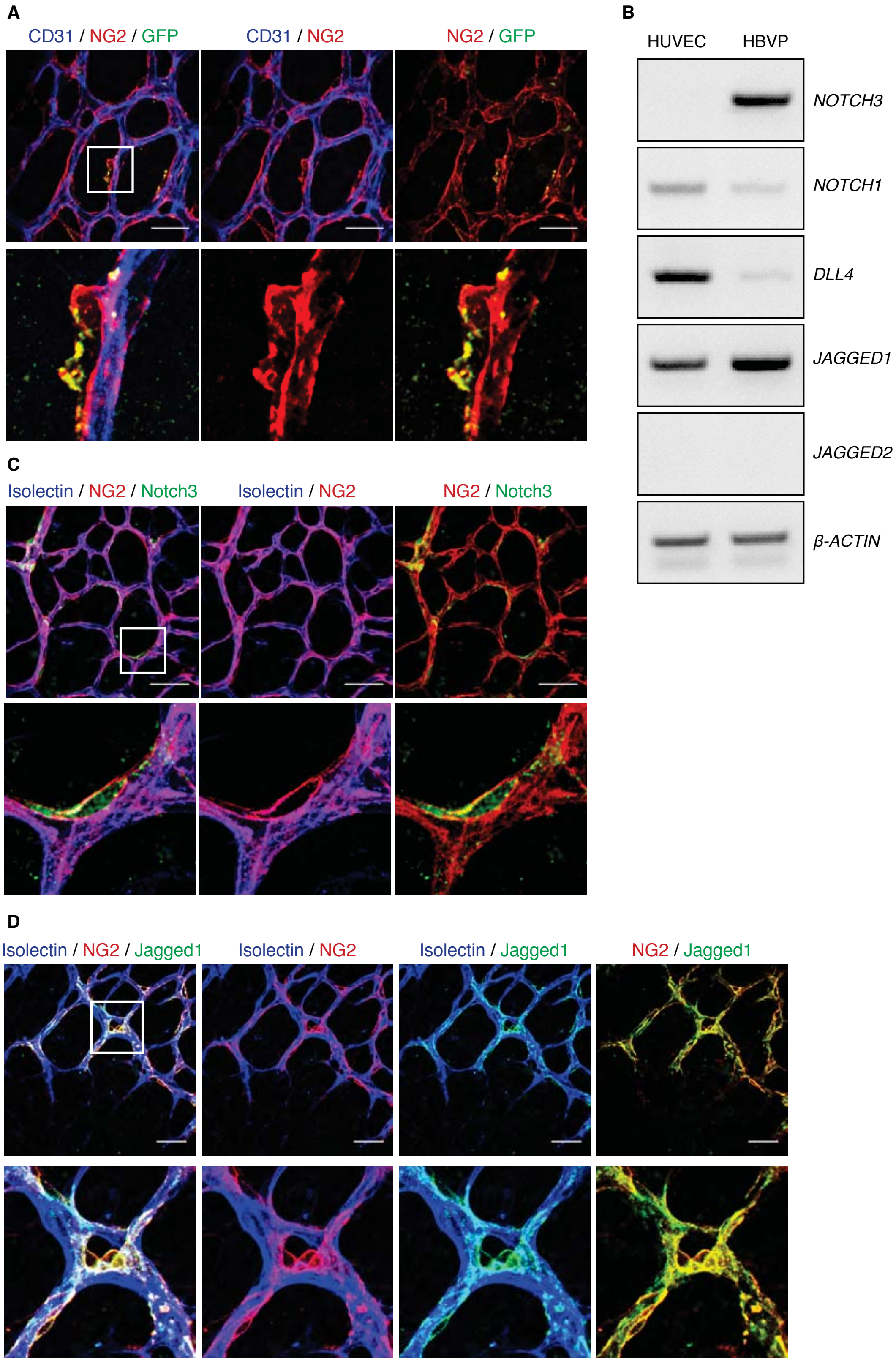

Supplemental Figure S1

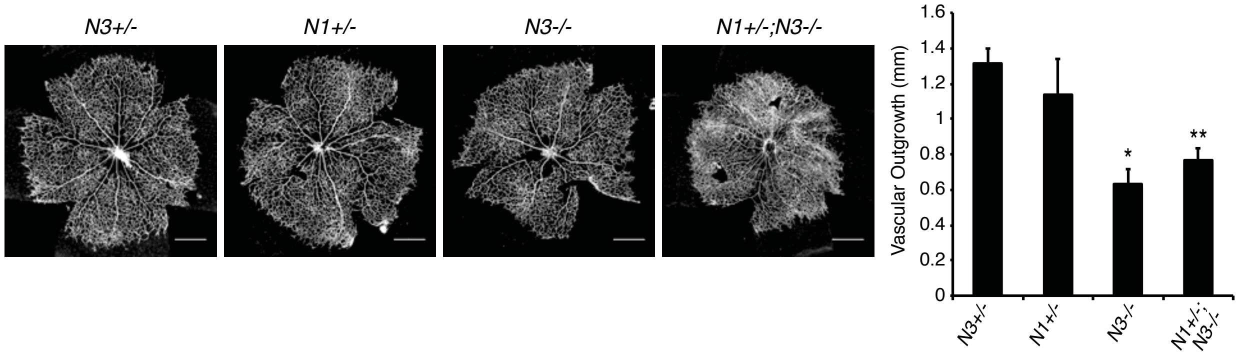

Supplemental Figure S2

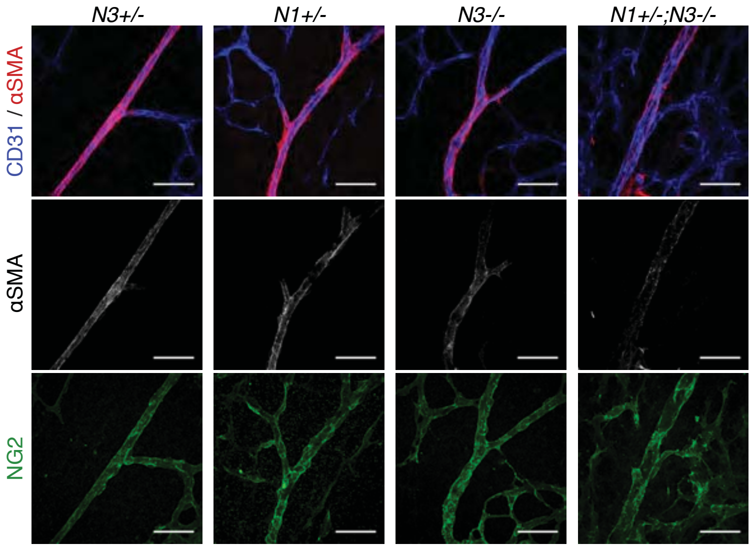

Supplemental Figure S3

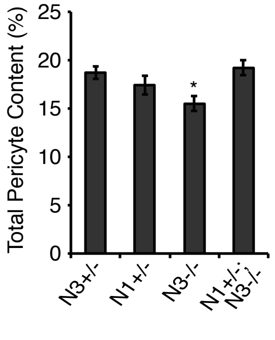

Supplemental Figure S4

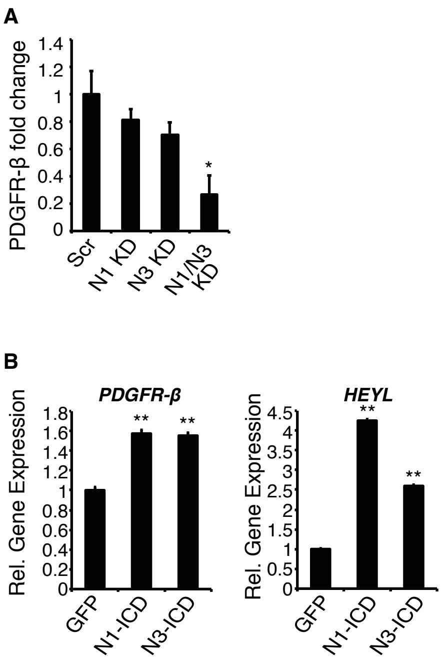

Supplemental Figure S5

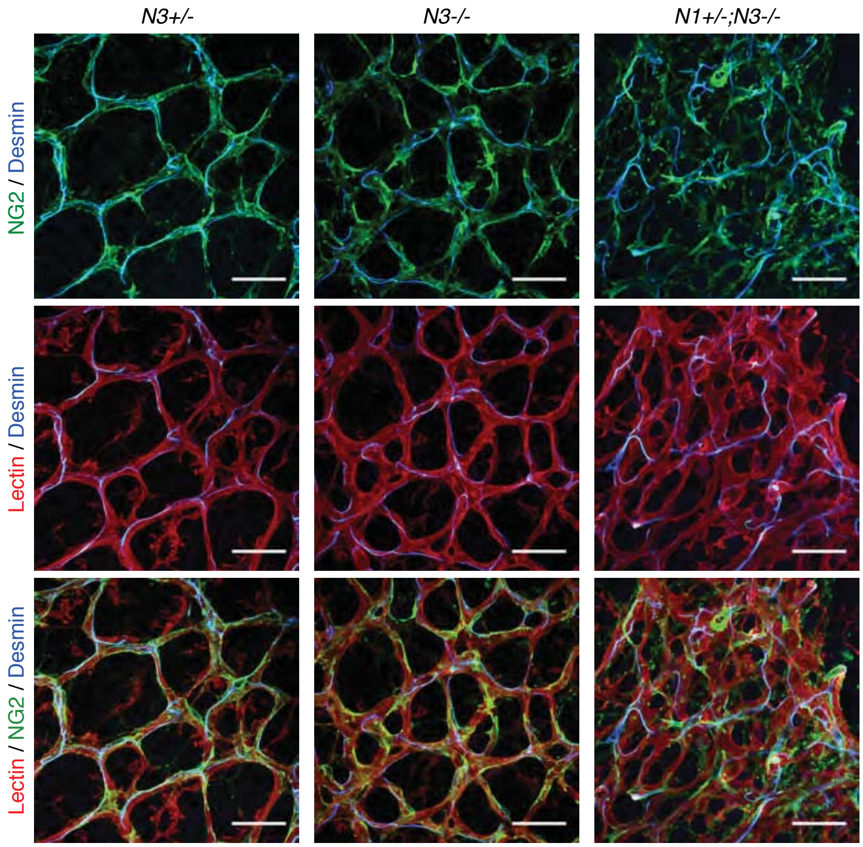

Supplemental Figure S6

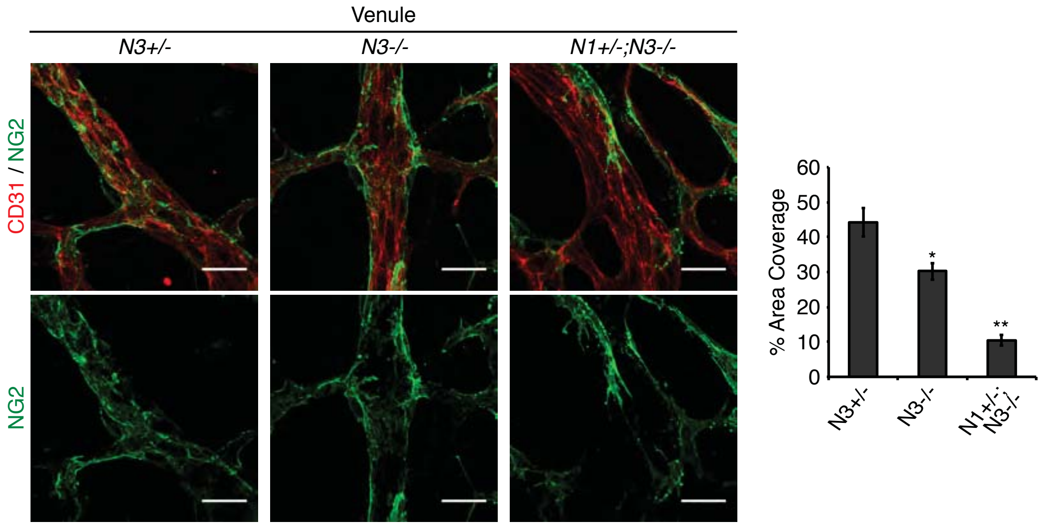

Supplemental Figure S7

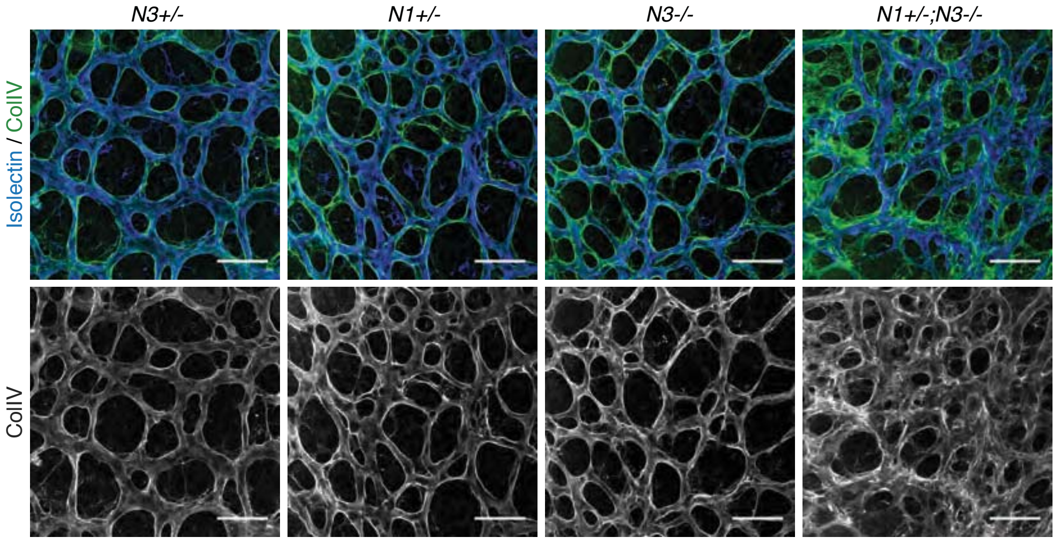

Supplemental Figure S8

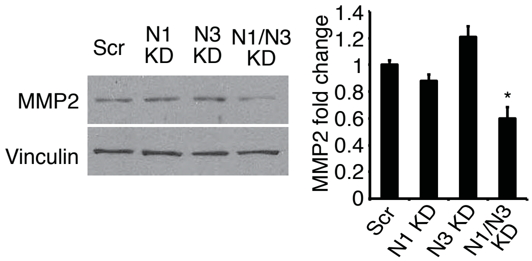

Supplemental Figure S9
